# Qualitative and Quantitative Analysis of ROS-Mediated Oridonin-Induced Oesophageal Cancer KYSE-150 Cell Apoptosis by Atomic Force Microscopy

**DOI:** 10.1371/journal.pone.0140935

**Published:** 2015-10-23

**Authors:** Jiang Pi, Huaihong Cai, Hua Jin, Fen Yang, Jinhuan Jiang, Anguo Wu, Haiyan Zhu, Jianxin Liu, Xiaohui Su, Peihui Yang, Jiye Cai

**Affiliations:** 1 State Key Laboratory of Quality Research in Chinese Medicines, Macau University of Science and Technology, Macau, China; 2 Department of Chemistry, Jinan University, GuangZhou, China; 3 Department of Pharmacology, Hunan University of Medicine, HuaiHua, China; LAAS-CNRS, FRANCE

## Abstract

High levels of intracellular reactive oxygen species (ROS) in cells is recognized as one of the major causes of cancer cell apoptosis and has been developed into a promising therapeutic strategy for cancer therapy. However, whether apoptosis associated biophysical properties of cancer cells are related to intracellular ROS functions is still unclear. Here, for the first time, we determined the changes of biophysical properties associated with the ROS-mediated oesophageal cancer KYSE-150 cell apoptosis using high resolution atomic force microscopy (AFM). Oridonin was proved to induce ROS-mediated KYSE-150 cell apoptosis in a dose dependent manner, which could be reversed by N-acetylcysteine (NAC) pretreatment. Based on AFM imaging, the morphological damage and ultrastructural changes of KYSE-150 cells were found to be closely associated with ROS-mediated oridonin-induced KYSE-150 cell apoptosis. The changes of cell stiffness determined by AFM force measurement also demonstrated ROS-dependent changes in oridonin induced KYSE-150 cell apoptosis. Our findings not only provided new insights into the anticancer effects of oridonin, but also highlighted the use of AFM as a qualitative and quantitative nanotool to detect ROS-mediated cancer cell apoptosis based on cell biophysical properties, providing novel information of the roles of ROS in cancer cell apoptosis at nanoscale.

## Introduction

Reactive oxygen species (ROS) within cells, such as hydrogen peroxide, superoxide anions and hydroxyl radicals, act as second messengers in the regulation of many important cellular events, including transcription factor activation, gene expression and cellular proliferation, differentiation and senescence [1]. ROS have also been implicated in the metabolic reprogramming of cancer cells, playing important roles in tumor initiation, progression, and metastasis [2]. And based on the different redox status of normal and cancer cells, a promising therapeutic strategy based on drugs that increase ROS generation and induce apoptosis in cancer cells comes out for cancer therapy [3]. High levels of ROS can directly induce oxidative damage in lipids, proteins and nucleic acids, therefore kill cancer cells by disturbing the metabolism and signal transduction. Increased ROS production is always involved in the anticancer mechanism of potential anticancer drugs, and also involved in some clinical used anticancer drugs, such as paclitaxel, 5-fluorouracil and doxorubicin [4–6].

Rabdosia rubescens, a kind of herbal medicine, has been traditionally used in China for the treatment of pharyngitis and esophageal carcinoma. Oridonin, the main pharmacological active substance of rabdosia rubescens with various pharmacological and physiological effects, has drawn a rising attention for cancer biologists due to its remarkable anti-tumor activities [7, 8]. It has been reported that oridonin can induce apoptosis or autophagy in various kinds of cancer cells, such as multiple myeloma cells [9], colorectal cancer cells [10], hepatoma carcinoma cell [11], prostate cancer cells [12], cervical carcinoma cells [13] and.oesophageal cancer cells [14]. And very interestingly, exposure of these cancer cells to oridonin results in a significant increase in ROS generation and the ROS scavenger, such as N-acetylcysteine (NAC), completely protects these cancer cells from oridonin induced cell death [9–13]. Therefore, oridonin could be served as an ideal anticancer agent for the study of ROS-mediated apoptosis in cancer cells.

As a member of scanning tunneling microscopy (STM) techniques, atomic force microscopy (AFM) is very useful in topography imaging, mechanical determination and single molecule force investigation relying on the detection of cantilever deflection induced by the forces between the AFM tip and sample. Based on these advantages, AFM has become one of the most powerful nanotechnologies for *in situ* single molecule imaging of cells, especially for cell membrane detections [15]. Recently, AFM has been introduced for the study of cancer cell death induced by drug treatment, which not only provides the high resolution morphological information, but also highlights the biomechanical changes during cell death [16–18]. These works demonstrate that AFM is very useful for the study of anticancer effects of drugs based on the cellular biophysical properties. Previous AFM studies have demonstrated that cancer cell apoptosis is closely related to the intracellular ROS level [19–21]. But there is still no systematic AFM study or analysis about the changes of biophysical properties in ROS-mediated cancer apoptosis.

In the present study, using high resolution AFM, we systematically investigated the biophysical properties of human oesophageal cancer KYSE-150 cells, which were found to be closely related to ROS-mediated apoptosis induced by oridonin. Oridonin was found to inhibit the proliferation, disrupt mitochondrial membrane potential and induce apoptosis of KYSE-150 cells all through the production of ROS in KYSE-150 cells. All these effects of oridonin on KYSE-150 cells could be reversed by the ROS-scavenger NAC, indicating the ROS-mediated anticancer effects of oridonin on KYSE-150 cells. Then, high resolution AFM was applied for the analysis of the anticancer effects of oridonin on KYSE-150 cells based on the biophysical properties of cells. And notably, we found that, for the first time, AFM detected morphological damage, membrane ultrastructural changes and biomechanical changes of apoptotic KYSE-150 cells induced by oridonin were also closely related to the intracellular ROS level of KYSE-150 cells, demonstrating the use of AFM as a powerful nanotool for the study of ROS-mediated cancer cell apoptosis.

## Materials and Methods

### Materials

Oridonin (≥98%, HPLC) is obtained from Mingwang biotechology (China). Fetal calf serum (FCS), penicillin/streptomycin, dulbecco’s modified eagle medium (DMEM), and trypsin kit are obtained from Gibco (USA). Paraformaldehyde is purchased from Sigma (USA). 3-(4, 5)-dimethylthiazo(-z-y1)-3,5-diphenyt- etrazoliumromide (MTT), N-acetyl-L-cysteine (NAC), Annexin V-FITC/PI (Annexin V-Fluorescein Isothiocyanate/Propidium Iodide) apoptosis detection kit, DCFH-DA (2',7'-dichlorodihydrofluorescein diacetate) reactive oxygen species assay kit, rhodamin 123, actin-tracker green (phalloidin- Fluorescein Isothiocyanate), 2-(4-amidinophenyl)-6-indolecarbamidine dihydrochloride (DAPI) and cell cycle analysis kits (Propidium Iodide) are purchased from Beyotime Institute of Biotechnology, China.

### Cell culture

Human oesophageal cancer KYSE-150 cell line is purchased from tumor cell library of Chinese Academy of Medical Sciences (BeiJing, China), which are cultured with DMEM medium supplemented with 10% FBS, 100 U/mL penicillin, and 100 g/mL streptomycin in a humidified atmosphere of 5% CO_2_ at 37°C.

### Cell viability measurement

MTT assay was used to test the cell viability of KYSE-150 cells exposed to oridonin. The cells were seeded into 96 well plates with a density of 5×10^3^ cells/well for 24 h and incubated with different concentration of oridonin for 24 h. To scavenge the ROS produced by oridonin, cells were pretreated with 2.5 mM NAC for 1 h and then treated with oridonin for 24 h. After oridonin treatment, MTT reagent (10 μL, 5 mg/mL) was then added into each well for 4 h incubation, the medium was removed, and the cells were suspended in 150 uL DMSO to incubate for 10 min. A spectrophotometer (TECAN, Switzer-land) was used to test absorbance at 490 nm.

### Intracellular ROS level determination

The intracellular ROS level of KYSE-150 cells was determined by a DCFH-DA based reactive oxygen species assay kit. The cells were seeded into 6 well plates with a density of 1×10^5^ cells/well for 24 h and incubated with different concentration of oridonin for 3 h. To scavenge the ROS produced by oridonin, cells were pretreated with 2.5 mM NAC for 1 h and then treated with oridonin for 3 h. After oridonin treatment, cells were harvested, washed triple with PBS and incubated with DCFH-DA solution for 30 min in dark at 37°C. Flow cytometry (BD, USA) was used to detect the intracellular ROS level after the cells were collected and washed twice with PBS.

### Cell apoptosis detection

Annexin V-FITC/PI apoptosis detection kit was used to detect the apoptosis of oridonin treated KYSE-150 cells according to the manufacturer’s instructions. The cells were seeded into 6 well plates with a density of 1×10^5^ cells/well for 24 h and incubated with different concentration of oridonin for 24 h. To scavenge the ROS produced by oridonin, cells were pretreated with 2.5 mM NAC for 1 h and then treated with oridonin for 24 h. After incubated with oridonin, KYSE-150 cells were harvested, washed triple with PBS, suspended in Annexin V binding buffer, and incubated with FITC-labeled Annexin V and PI for 5 min at room temperature in dark. Then, the samples were immediately analyzed by Flow cytometry (BD, USA).

### Mitochondrial membrane potential analysis

Rhodamine 123-based flow cytometry was used to determine the alterations of mitochondrial membrane potential (MMP) of KYSE-150 cells upon oridonin treatment. The cells were seeded into 6 well plates with a density of 1×10^5^ cells/well for 24 h and incubated with different concentration of oridonin for 24 h. To scavenge the ROS produced by oridonin, cells were pretreated with 2.5 mM NAC for 1 h and then treated with oridonin for 24 h. The harvested and washed KYSE-150 cells were incubated with rhodamine 123 for 60 min in dark at 37°C. Flow cytometry (BD, USA) was used to detect the fluorescence signal of rhodamine 123 after the cells were collected and washed twice with PBS.

### AFM sample preparation

For AFM measurements, KYSE-150 cells were harvested with 0.25% trypsin and cultured at a density of 5 × 10^4^ cells/well on glass coverslips in a six well plate. After overnight incubation, oridonin was added into the culture medium for 24 h stimulation. To scavenge the ROS produced by oridonin, cells were pretreated with 2.5 mM NAC for 1 h and then treated with oridonin for 24 h. After that, cells were washed triple with PBS, fixed with 4% paraformaldehyde solution for 10 min, washed triple with distilled water and dried in air for morphology imaging in air or immediately used for force measurements in PBS solution. For living cell stiffness measurements, KYSE-150 cells were treated with or without oridonin and NAC for 24 h, and then washed with PBS buffer to discard the suspending cells in medium. The cells were then used for Young’s modulus measurements in DMEM medium by AFM.

### AFM morphology imaging and membrane ultrastructure analysis

AFM was used to investigate the topographical and ultrastructural (Bruker, German) changes of KYSE-150 cells induced by oridonin treatment. Organic contaminates of the silicon nitride tips used in all measurements were removed by ultraviolet irradiation. The spring constants of the AFM tips were calibrated using the thermal noise method implemented in the Nanoscope software on AFM. Firstly, the calibration of deflection sensitivity was carried out on the glass cover slips at a small vertical deflection (~ 0 V) in air. Then the thermal tune curve was fitted by the simple harmonic oscillator fitting to calculate the spring constant. The curvature radius of the AFM tips (BudgetSensors, Bulgaria) used for morphology imaging was 10 nm, and the spring constant of tips was 0.51±0.02 N/m with a deflection sensitivity of 52.94±0.96 nm/V. The morphological and ultrastructural images of KYSE-150 cells were obtained in air at room temperature in contact mode. The ultrastructure of KYSE-150 cells was obtained in the areas surrounding the nuclei and the topographical image processing and data analysis were performed using the instrument equipped Nanoscope Analysis software. And to make the scale bar, X axis labels and Y axis labels of AFM images more readable, we re-writed the scale bar and labels for X and Y axis in these Figures. For the ultrastructure parameter analysis, 30 different 2×2 um^2^ ultrastructure images on 15 different KYSE-150 cells in each group were calculated. Before ultrastructure parameter analysis, AFM images were flattened with the first flatten order by Nanoscope Analysis software. After that, the whole ultrastructure image was calculated for height distribution and roughness by Nanoscope analysis software.

### AFM stiffness analysis

Biomechanical properties of living KYSE-150 cells were measured in DMEM medium by bare tips in Force Volume mode through AFM (Bruker, German). The spring constant of silicon nitride probes used for living cell measurements (Bruker, German) was calibrated by the thermal-noise method in a clean culture dish containing PBS, which was 0.08±0.01 N/m with a deflection sensitivity of 16.23±0.71 nm/V. In the Force Volume mode, the tip was alternately approached to cell surface and then retracted from 16×16 points over 1×1 um^2^ area on sample surface while force curves were synchronously recorded. For living cell meaurements, more than 15 different locations on 15 different cells were recorded at the central area of cells in each group. The Young’s modulus was calculated from the force curves by basic Sneddon model, which described the behavior of a known geometry indenter in contact with an elastic half-space much less rigid than the punch as shown in Eq (1) [22]:

$$\text{F}=\frac{2}{\pi }\text{tan}\left(\alpha \right)\frac{E}{\left(1-\nu^{2}\right)}\delta^{2}$$ (1)

Where υ, F, δ, E, and α are Poisson ratio, loading force, indentation, Young’s modulus, and the half-opening angle of a conical tip, respectively. In Sneddon model, the load exerted by the punch is linked to the caused indentation depth, which shows the relationship between the applied force F and the indentation δ in the case of conical indenter. A Poisson ratio of 0.5 is appropriate for cells and thus used in the following analysis [23, 24].

### Statistics

All results are representative of three independent experiments and the data presented are expressed as Mean ± S.D.. Statistical analysis was performed using Student’s t-test except that the Young’s modulus is performed using Kruskal-Waillis test, and p < 0.05 was regarded as statistically significant.

## Results and Discussion

### Oridonin inhibit KYSE-150 cell viability by ROS-mediated pathway

**Fig 1:**
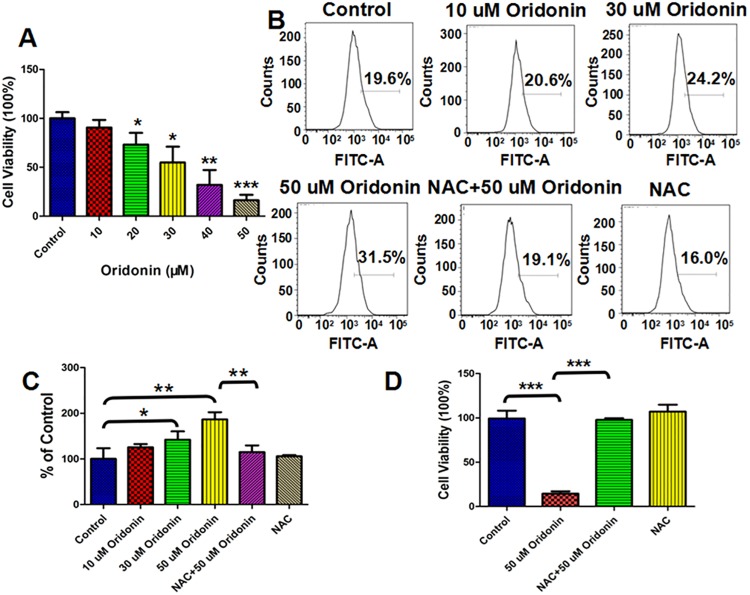
ROS scavenger-NAC reversed oridonin inhibited cell proliferation and oridonin induced intracellular ROS production in oesophageal cancer KYSE-150 cells. (A) Effects of oridonin on the viability of KYSE-150 cells. (B) DCFH-DA assay of the effects of NAC on oridonin induced ROS production in KYSE-150 cells. (C) Statistical analysis of the effects of NAC on oridonin induced ROS production in KYSE-150 cells. (D) Effects of ROS scavenger-NAC on oridonin inhibited viability of KYSE-150 cells, *p<0.05, **p<0.01, ***p<0.001.

Oridonin has been found to show broad-spectrum anticancer activities against different cancer cell lines [9–13, 25]. The anti-proliferation effects of oridonin against human oesophageal cancer KYSE-150 cells were investigated by MTT assay, which indicated strong proliferation inhibition effects of 24 h oridonin treatment on KYSE-150 cells as shown in Fig 1A. The viability of KYSE-150 cells decreased from 100.0±6.3% to 90.6±7.8%, 73.2±11.9%, 54.8±16.3%, 32.0±15.3% and 16.2±5.6% after 10 μM, 20 μM, 30 μM, 40 μM and 50 μM oridonin treatment, respectively (Fig 1A). The previous works focused on the anticancer activity of oridonin implied that the inhibition effects of oridonin were always accompanied by the increase of intracellular ROS level in cancer cells [9–13]. We also determined the ROS level in KYSE-150 cells, which demonstrated that oridonin treatment could significantly induce the over-production of ROS in a dose dependent manner and the over-produced ROS induced by oridonin could be eliminated by the pre-treatment of cells with ROS scavenger NAC (Fig 1B). The relative intracellular ROS level in KYSE-150 cells increased from 100.0±23.4% for control cells to 125.5±6.9%, 142.1±18.2% and 186.6±15.8% for 10 μM, 30 μM and 50 μM oridonin treated cells, respectively (Fig 1C). With the pretreatment of 2.5 mM NAC for 1 h, the intracellular ROS level in KYSE-150 cells upon 50 μM oridonin treatment decreased to 114.9±14.8% and the pretreatment with NAC alone showed no significant effects on the ROS level in KYSE-150 cells with an average ROS level of 105.6±2.8% (Fig 1C). These results implied that oridonin could induce the over-production of ROS in KYSE-150 cells, which could be eliminated by NAC pretreatment.

To determine the roles of ROS in the anticancer activity of oridonin, we also detect the effects of NAC on oridonin inhibited KYSE-150 cell proliferation. As shown in Fig 1D, the viability of KYSE-150 cells was 14.6±2.5% after 50 μM oridonin treatment, which was reversed to be 97.8±1.9% by the 1 h pretreatment with 2.5 mM NAC. These results strongly suggest the inhibition effects of oridonin on KYSE-150 cell proliferation in ROS-mediated pathway.

### Oridonin-induced ROS-mediated KYSE-150 cell apoptosis

**Fig 2:**
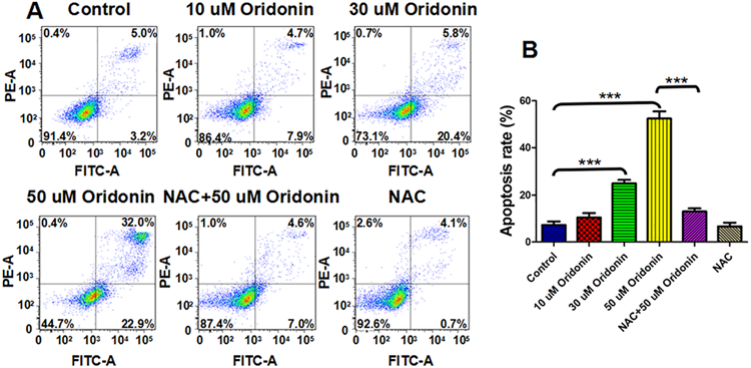
ROS scavenger-NAC reversed oridonin induced oesophageal cancer KYSE-150 cell apoptosis. (A) Annexin V/PI assay of the effects of NAC on oridonin induced KYSE-150 cell apoptosis. (B) Statistical analysis of the effects of NAC on oridonin induced KYSE-150 cell apoptosis, ***p<0.001.

Induction of cancer cell apoptosis has been widely studied in recent decades, making it one of the most common and important indicators that need to be detected for the development of anticancer drugs. To further clarify the anticancer activity of oridonin in human oesophageal cancer cells, the apoptosis of KYSE-150 cells was determined by Annexin V-FITC/PI assay. As indicated in Fig 2A, oridonin could induce apoptosis of KYSE-150 cells in a dose dependent manner, and the pretreatment with NAC could inhibit oridonin induced KYSE-150 cell apoptosis. The apoptosis rate of KYSE-150 cells increased from 7.3±1.5% for control cells to 10.5±1.9%, 24.8±1.5% and 52.4±3.1% after 10 μM, 30 μM and 50 μM oridonin treatment, respectively (Fig 2B). And with the pretreatment of NAC, the apoptosis rates of KYSE-150 cells were 13.0±1.3% and 6.7±1.6% with and without 50 μM oridonin treatment, respectively (Fig 2B). Oridonin induced apoptosis of KYSE-150 cells was mostly all reversed by NAC treatment, which demonstrated that oridonin induced KYSE-150 cell apoptosis was closely related to the over-produced ROS induced by oridonin.

**Fig 3:**
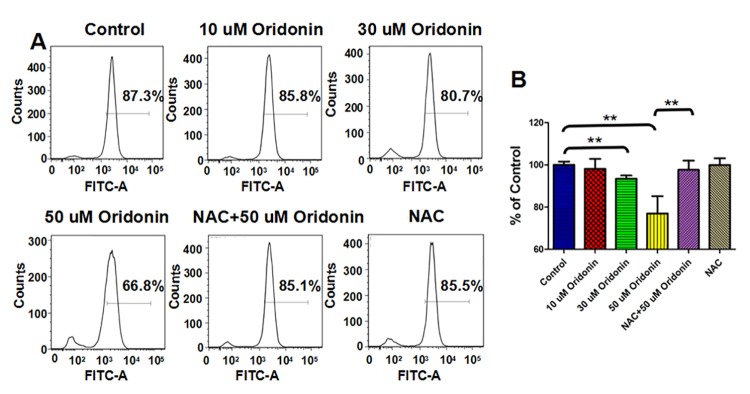
ROS scavenger-NAC reversed oridonin induced disruption of mitochondrial membrane potential in oesophageal cancer KYSE-150 cells. (A) Rhodamine 123 assay of the effects of NAC on oridonin induced disruption of mitochondrial membrane potential in KYSE-150 cells. (B) Statistical analysis of the effects of NAC on oridonin induced disruption of mitochondrial membrane potential in KYSE-150 cells, **p<0.01.

To determine if the dysfunction of mitochondria was also involved in oridonin induced KYSE-150 cell apoptosis, we examined the mitochondrial membrane potential (MMP) in response to oridonin exposure by flow cytometry using rhodamine 123 as a fluorescence probe. The results implied that oridonin could induce MMP disruption of KYSE-150 cells in a dose dependent manner, and NAC could also reverse this effect (Fig 3A). The statistical analysis showed that the relative fluorescence signal of rhodamine 123 decreased from 100.0±1.6% for control cells to 98.1±4.7%, 93.4±1.5% and 77.0±8.2% after 10 μM, 30 μM and 50 μM oridonin treatment, respectively (Fig 3B). The pretreatment with NAC had no significant effects on the MMP of KYSE-150 cells, which showed a relative value of 99.96±1.82% (Fig 3B). And the relative fluorescence signal of rhodamine 123 also increased to 97.7±4.4% with the pretreatment with NAC and treatment with 50 μM oridonin, indicating that oridonin could also induce MPP disruption of KYSE-150 cells by ROS-mediated way. Additionally, the analysis of cell cycle distribution also demonstrated that oridonin could also induce cell cycle arrest of KYSE-150 cells at G2/M phase (Fig A in S1 File). Although the rates of cells at G2/M phase were not ROS level dependent, but the pretreatment with NAC could also reverse oridonin induced cell cycle arrest (Fig A in S1 File).

The treatment of oridonin significantly increased the ROS level in KYSE-150 cells, and the over-produced ROS further induced the disruption of MMP, the arrest of cell cycle and the apoptosis of KYSE-150 cells. With the pretreatment of NAC, which had no significant effects on the physiological status of KYSE-150 cells but could remove the over-produced ROS, oridonin induced MMP disruption, cell cycle arrest and apoptosis of KYSE-150 cells were dramatically reversed. These results strongly suggested that the anticancer activities of oridonin in KYSE-150 cells were mainly mediated by the intracellular ROS level of KYSE-150 cells induced by oridonin.

### ROS-dependent morphological damage of KYSE-150 cells

In recent years, AFM has emerged as a powerful tool for nanoscale morphology imaging and pico-newton sensitivity force measurement of cells in different physiological status [26–29]. The super resolution and high force sensitivity of AFM also show very wide applications for the detection of cancer cell apoptosis upon drug treatment, providing some novel information about cell morphology, cell biomechanics and membrane receptor functions [16, 18, 28]. The morphology of cells is closely related to cellular physiological status and functions. Cell apoptosis is always associated with a lot of typical morphological changes, such as condensation of nucleus, blebbing of the membrane and shrink of cell body.

**Fig 4:**
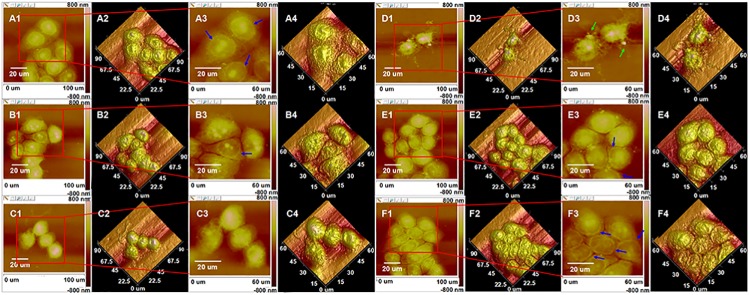
ROS scavenger-NAC reversed oridonin induced cell morphology damage of oesophageal cancer KYSE-150 cells. AFM morphology imaging of (A) control, (B) 10 μM oridonin treated, (C) 30 μM oridonin treated, (D) 50 μM oridonin treated, (E) 2.5 mM NAC+50 μM oridonin treated and (F) 2.5mM NAC treated KYSE-150 cells. (A1-F1) Topography images and (A2-F2) their corresponding 3D images of KYSE-150 cells; (A3-F3) Enlarged topography images in (A1-F1) and (A4-F4) their corresponding 3D images of KYSE-150 cells, scale bar: 20 μm.

To resolve the precise morphological changes of KYSE-150 cells induced by oridonin treatment and the roles of ROS in oridonin induced morphological changes of KYSE-150 cells, high resolution AFM was operated for the study of KYSE-150 cells. As shown in Fig 4A, control KYSE-150 cells showed typical oval shapes and the cells closely contacted with other cells. After treated with 10 μM oridonin for 24 h, there were no remarkable changes in the morphology of KYSE-150 cells (Fig 4B). After 24 h treatment with 30 μM oridonin, KYSE-150 cells showed some typical morphological characteristics of apoptosis, including the shrunk cell bodies and the condensed cytoplasm (Fig 4C). And for 50 μM oridonin treated KYSE-150 cells, more typical morphological characteristics of apoptosis could be observed, such as the shrink of cell bodies, condensation of cytoplasm, condensation and fragmentation of nucleus, the emergence of apoptotic body (Indicated by green arrows) and the damaged pseudopodium structures for cell connections (Fig 4D). To demonstrate the role of increased intracellular ROS level in oridonin induced morphological changes of KYSE-150 cells, the morphology of KYSE-150 cells with the pretreatment of 2.5 mM NAC for 1 h and 50 μM oridonin for 24 h was also investigated by AFM. The AFM images (Fig 4E) implied that KYSE-150 cells had no significant morphological changes upon 50 μM oridonin treatment due to the pretreatment of 2.5 mM NAC. And the pretreatment of NAC also had no remarkable effects on the morphology of KYSE-150 cells (Fig 4F). Additionally, we also found that control KYSE-150 cells always showed clear nucleus areas (Indicated by blue arrows in Fig 4), which also disappeared after oridonin treatment and reversed by the pretreatment with NAC. These high resolution AFM images suggested that oridonin could induce morphological damages of KYSE-150 cells and these morphological damages could also be reversed by NAC pretreatment, indicating that AFM can be used as a qualitative nanotool to determine ROS-mediated cell morphological changes in cancer cell apoptosis. However, this morphological imaging method based on AFM is restricted to the qualitative analysis of ROS-mediated cancer cell apoptosis, and isn’t suitable for quantitative analysis.

### ROS-dependent membrane ultrastructural changes of KYSE-150 cells

**Fig 5:**
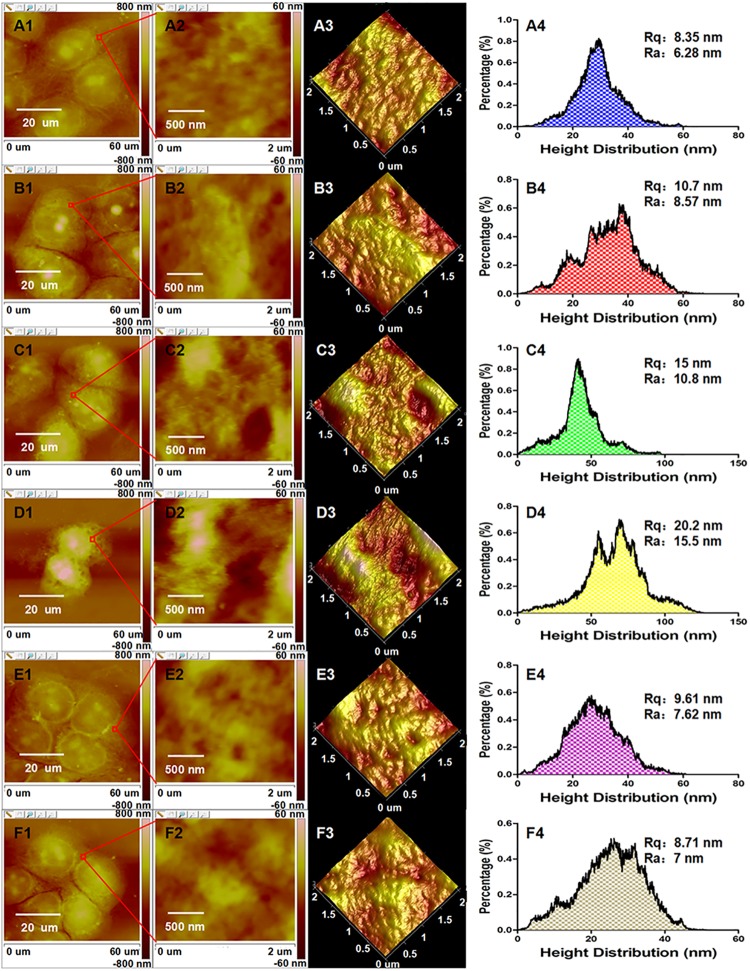
ROS scavenger-NAC reversed oridonin induced oesophageal cancer KYSE-150 cell membrane ultrastructural changes. AFM morphology and membrane ultrastructure imaging of (A) control, (B) 10 μM oridonin treated, (C) 30 μM oridonin treated, (D) 50 μM oridonin treated, (E) 2.5 mM NAC+50 μM oridonin treated and (F) 2.5mM NAC treated KYSE-150 cells. (A1-F1) Topogrphy images of KYSE-150 cells, scale bar: 20 μm; (A2-F2) Enlarged membrane ultrastructure images in (A1-F1) and (A3-F3) their corresponding 3D images of KYSE-150 cells, scale bar: 500 nm; (A4-F4) Height distribution and roughness of cell surface ultrastructure analyzed from (A2-F2).

Cell membrane ultrastructure was also widely used to evaluate the apoptosis of cancer cells by AFM because the changes of membrane components, such as lipid raft, membrane proteins and phospholipid, were also associated with cell apoptosis [16, 18, 28, 30, 31]. With the high resolution imaging of AFM, some parameters describing the property of cell membrane could be extracted from the AFM ultrastructure images to clarify the changes of cell membrane during apoptosis. The effects of oridonin on the membrane ultrastructure of KYSE-150 cells were also determined by AFM analysis to investigate the more detailed morphological changes of oridonin induced KYSE-150 cell apoptosis. By enlarged imaging of cell membrane around the nucleus areas, the membrane ultrastructural parameters could be extracted for quantitative analysis of oridonin induced KYSE-150 cell apoptosis. As shown in Fig 5, changes of membrane structure were difficult to clarify by the direct observation of membrane ultrastructure images of KYSE-150 cells. As shown in Fig 5A4–5F4, the height distribution on cell membrane and the roughness of cell membrane (Including Rq and Ra) increased after oridonin treatment, which were then reversed with the pretreatment of NAC.

**Fig 6:**
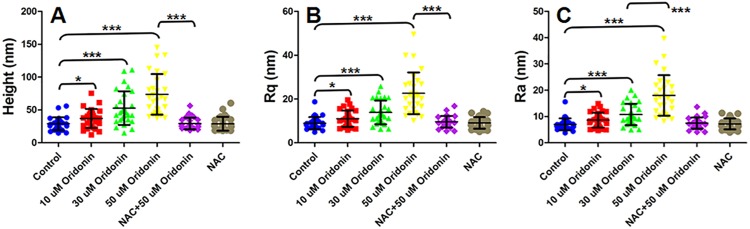
Statistical results of NAC reversed oridonin induced oesophageal cancer KYSE-150 cell membrane ultrastructural changes determined by AFM. (A) Height distribution, (B) root-mean-squared roughness (Rq) and (C) average roughness (Ra) analyzed from 2×2 μm frame ultrastructure images of KYSE-150 cells, n = 30, *p<0.05, ***p<0.001.

By calculating 30 different locations in 15 different cells in each group, the average height, Rq (root-mean-squared roughness) and Ra (average roughness) were presented in Fig 6. The average height of KYSE-150 cell membrane increased from 28.9±9.9 nm for control cells to 37.1±14.5 nm, 52.7±25.7 nm and 73.7±31.0 nm for 10 μM, 30 μM and 50 μM oridonin treated KYSE-150 cells, respectively (Fig 6A). The dramatic increase of membrane partcle size in KYSE-150 cells after 50 μM oridonin treatment could also be eliminated with the pretreatment of NAC, which showed an average height of 29.3±9.0 nm (Fig 6A). And the average height of NAC treated KYSE-150 cells was 29.0±10.5 nm, showing no significant effects on the membrane height distribution of KYSE-150 cells (Fig 6A). The Rq values of KYSE-150 cells also increased, from 8.9±2.8 nm for control cells to 10.99±0.69 11.0±3.7 nm, 13.87±1.02 13.9±5.4 nm and 22.6±9.5 nm for 10 μM, 30 μM and 50 μM oridonin treated KYSE-150 cells, respectively (Fig 6B). And if the cells were pretreated with NAC, the Rq values were 9.6±2.7 nm and 9.1±2.7 nm with and without 50 μM oridonin treatment, respectively (Fig 6B). Similarly, the Ra value was 7.1±2.3 nm for control cells, and increased to 8.6±2.8 nm, 10.7±4.1 nm and 18.0±7.7 nm after 10 μM, 30 μM and 50 μM oridonin treatment, respectively (Fig 6C). The significant increase of Ra in KYSE-150 cells after 50 μM oridonin treatment could also be reversed with the pretreatment of NAC to show a Ra value of 7.5±2.1 nm (Fig 6C). And the NAC pretreated KYSE-150 cells without oridonin treatment showed a Ra value of 7.2±2.1 nm (Fig 6C). These results indicated that, similar with the intracellular ROS level, both the membrane height distribution and membrane roughness of KYSE-150 cells increased after oridonin treatment in a dose dependent manner, and the increased membrane height distribution and membrane roughness could also be reversed by NAC pretreatment.

It is well known that cell membrane ultrastructure is influenced by several environmental and subcellular state factors such as motility, adhesion, and intracellular contact, thus could be developed into very good indicators of the health or unnormal status of cells [32]. The height distribution and roughness of cell membrane determined by AFM are parameters describing the structure of cell membrane. The increased membrane height distribution could be attributed to the aggregation of membrane biomolecules. As reported, the hole like structures would lead a rougher surface of cells [18]. And generally, more molecules located on plasma membranes, larger roughness of cell surface would be detected by AFM [33]. Cell apoptosis is associated with the exposure of inner membrane phospholipid at the cell surface, which thus produces integrated changes of electrostatic potential and hydration in the outer leaflet of cell membrane [34]. And in ROS-mediated apoptosis, high intracellular ROS levels are also found to affect the structure or expression of some important membrane components, such as transmembrane channels, receptors and lipid raft [35–37]. The results had demonstrated that oridonin could induce the over-production of ROS to further induce KYSE-150 cell apoptosis in a dose-dependent manner (Figs 1 and 2), and the increase of intracellular ROS level might also have strong effects on the membrane structure of KYSE-150 cells. Our results demonstrated that the ROS-dependent apoptosis of KYSE-150 cells induced by oridonin could also be quantitatively detected by AFM ultrastructure imaging and analysis, providing evidences for the dose dependent damage of cell membranes. And the pretreatment with NAC could also reverse the membrane ultrastructural changes of KYSE-150 cells induced by oridonin, demonstrating that the membrane ultrastructural damages were also ROS dependent. Based on high resolution AFM imaging, the cell surface ultrastructre is visulized and quantified, which could be further used to detect ROS-dependent cancer cell apoptosis. This method provides some novel information about the cell membrane ultrastructure in ROS-mediated cell apoptosis.

### ROS-dependent cellular biomechanical changes of KYSE-150 cells

Despite the high resolution morphological imaging, AFM is also very useful to quantitatively measure the biomechanics of cells with pico-newton sensitivity using its force measurement function. Cell biomechanics determined by AFM, such as adhesion force and stiffness, are found to be closely related to some important physiological functions and the health status of cells [26–29]. In recent decades, the biomechanical properties of living or fixed cells have been widely investigated to understand the roles of biomechanics in cell functions [16, 23, 24, 38–40]. For AFM force measurement, the AFM tip is located onto the surface of cell samples, presses against the sample and then retracts from the cell surface. During the retraction process of AFM tip, the retraction results occur as adhesive events between the tip and cell membrane. Based on these adhesive events, the adhesion force between AFM tips and cell surface can be determined, and the stiffness of cell sample could also be further analyzed. The adhesion force of cells determined by AFM are induced by the interactions between the AFM tip and cell membrane, which could be served as a parameter describing the adhesion and spreading of cells. We investigated the adhesion force of KYSE-150 cells upon oridonin treatment, the increased adhesion force was found in oridonin treated KYSE-150 cells (Fig B in S1 File). But the increased adhesion force for 50 μM oridonin treated cells was much smaller than that of 30 μM oridonin treated cells, indicating that the adhesion force detected by AFM was not positively correlated with cell apoptosis. Generally, the adhesion force between the cell surface and an AFM tip is attributed to the amount of carbohydrate-containing molecules or adhesion molecules on plasma membranes[41]. Therefore, the non dose dependent increase of adhesion force in oridonin induced KYSE-150 cells might be controlled by the amount of carbohydrate-containing molecules or adhesion molecules on cell membrane.

**Fig 7:**
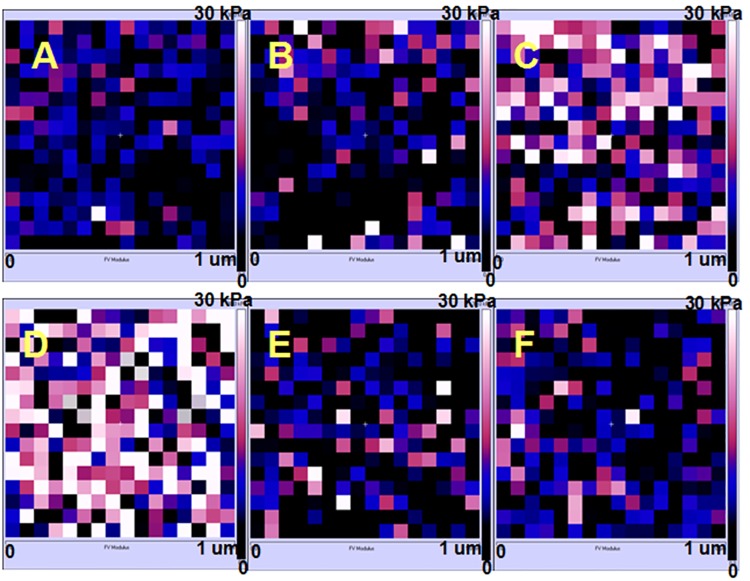
ROS scavenger-NAC reversed oridonin induced changes of Young’s modulus in oesophageal cancer KYSE-150 cells. Typical Young’s modulus maps obtained from (A) control, (B) 10 μM oridonin treated, (C) 30 μM oridonin treated, (D) 50 μM oridonin treated, (E) 2.5 mM NAC+50 μM oridonin treated and (F) 2.5mM NAC treated KYSE-150 cells.

**Fig 8:**
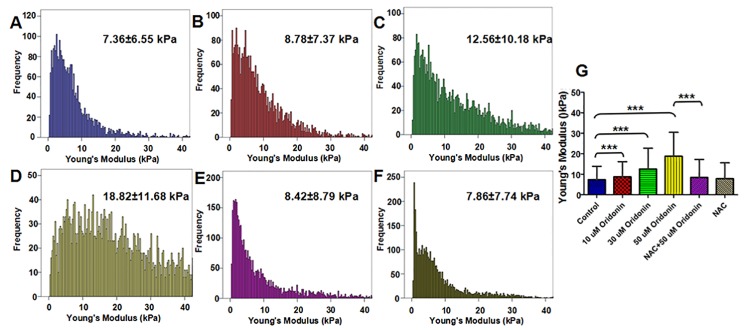
ROS scavenger-NAC reversed oridonin induced changes of Young’s modulus in oesophageal cancer KYSE-150 cells. (A) Histogram distribution of Young’s modulus obtained from KYSE-150 cell. (B) Statistical analysis of the effects of NAC on oridonin induced KYSE-150 cell Young’s modulus changes, n>5000, ***p<0.001.

The stiffness of cancer cells can be served as an important indicator of the invasion or metabolism state of tumors [42, 43]. And, the measurements of stiffness in cancer cells are also of vital importance to understand the biophysical properties of cancer cells upon drug treatment [16, 18, 28]. To determine whether AFM based force measurement could also be used to detect ROS-mediated cell apoptosis, we further investigated the Young’s modulus (a parameter describing stiffness) of KYSE-150 cells by AFM using the basic Hertz-Sneddon model (the typical fitting processes of the force curve by Hertz-Sneddon model was shown in Fig C in S1 File). The Force Volume mode of AFM was applied for Young’s modulus analysis of living KYSE-150 cells to provide the Young’s modulus maps as shown in Fig 7. The representative color based Young’s modulus maps of KYSE-150 cells clearly indicated that the stiffness of KYSE-150 cells increased with the treatment of oridonin in a dose dependent manner, and the increased stiffness was reversed with the pretreatment of NAC. The histogram distribution analysis of Young’s modulus results (Fig 8) showed that the Young’s modulus was increased from 7.36±6.55 kPa for control living KYSE-150 cells to 8.78±7.37 kPa, 12.56±10.18 kPa, 18.82±11.68 kPa for 10 μM, 30 μM and 50 μM oridonin treated KYSE-150 cells, respectively. And if the cells were pretreated with NAC, the Young’s modulus values were 8.42±8.79 kPa nm and 7.86±7.74 kPa with and without 50 μM oridonin treatment, respectively. Additionally, we also determined the stiffness of fixed KYSE-150 cells (Fig D in S1 File), which showed similar changes with oridonin and NAC treatment. These AFM results suggested that oridonin treatment could induce KYSE-150 cells to be much stiffer, which could be reversed by the pretreatment of NAC.

Stiffness is one of the mechanical parameters describing the relation between an applied, nondestructive load and resultant deformation of a sample, which is very sensitive to the internal structural details of heterogeneous sample. In theory, the Young’s modulus calculated on cell surface is dependent on the intracellular structures of cells, including the strength of cytoskeleton and the presence of underlying organelles under cell surface, such as nuclei and mitochondria. A larger increase of stiffness, such as five-fold increasing, is always supposed to be mainly caused by the changes of underlying organelle distributions, whereas small and gradual increases of stiffness are mainly attributed to the hardening of cytoskeleton structures[44]. The gradual increase of stiffness in KYSE-150 cells induced by oridonin treatment indicated that the cytoskeleton structure changes might also be involved in the anticancer activities of oridonin against KYSE-150 cells.

As reported, the cytoskeleton structure of cells was always disrupted during the apoptosis of cells and the high intracellular ROS level also could induce oxidative damage of cytoskeleton structure of cells [45, 46]. As the most important part of cytoskeleton, the integrity of F-actin network was regarded as an important indicator of cell stiffness, which could be measured by AFM [47]. To clarify whether cytoskeleton was the main source of stiffness changes in KYSE-150 cells, we further investigated the structure of F-actin in KYSE-150 cells upon oridonin treatment by fluorescence imaging. The results demonstrated that oridonin treatment indeed induce the damage of F-actin structure in KYSE-150 cells, making thread-like F-actin fibres disappear and the close contacted F-actin between cells much weaker (Fig E in S1 File). And the pretreatment with NAC could also reverse the damage of F-actin in KYSE-150 cells induced by oridonin treatment (Fig E in S1 File). In addition, we also found that oridonin could induce the damage of nucleus of KYSE-150 cells, which could also be protected by the pretreatment of NAC. Based on these results, we could consider that the high intracellular ROS level induced by oridonin in KYSE-150 cells had strong destructive effects on the F-actin cytoskeleton structure of KYSE-150 cells, and these effects would be inhibited by removing the over-produced ROS with pretreatment of NAC.

**Fig 9:**
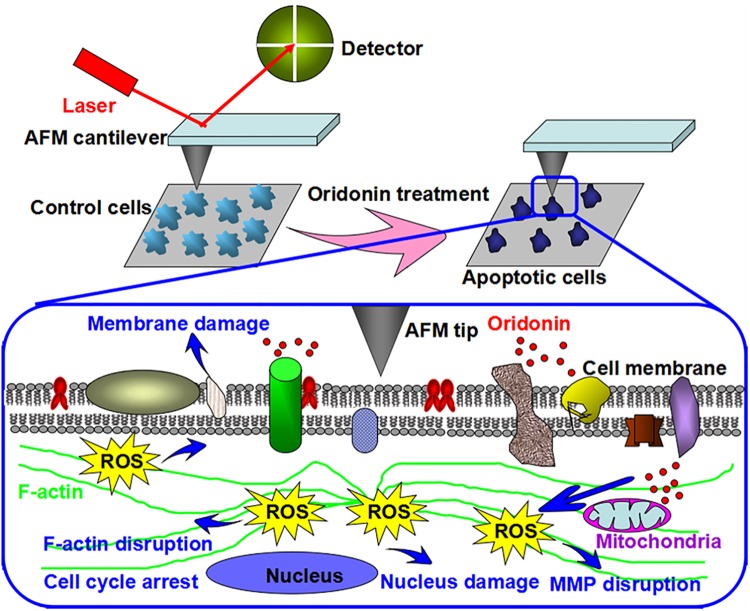
Schematic diagram showing the ROS-mediated oridonin induced KYSE-150 cell apoptosis that can be detected by AFM.

Taking all results obtained here into account, we proposed a schematic diagram of ROS-mediated-oridonin induced KYSE-150 cell apoptosis that could be qualitatively and quantitatively detected by AFM (Fig 9). Oridonin treatment could induce the over-production of ROS in KYSE-150 cells, which played a key role in oridonin induced KYSE-150 cell apoptosis. The over-produced ROS had strong destructive effects on the structure and function of mitochondria, which induced the disruption of mitochondrial membrane potential. The high intracellular ROS level also induced oxidative damage in the membrane component of KYSE-150 cells, which would disturb the native membrane structure of KYSE-150 cells. Based on the changes of cell membrane, high resolution AFM imaging could be applied for the membrane ultrastructure detection, which found the increased height and increased roughness in the membrane of ROS-mediated oridonin-induced apoptotic KYSE-150 cells. The over-produced ROS also showed toxicity in the nucleus of KYSE-150 cells, and then induce the damage of nucleus structure and the arrest of cell cycle. Additionally, the high ROS level also showed strong destructive effects on the cytoskeleton component F-actin, which induced the disruption of F-actin structure. On the one hand, the ROS induced damage of nucleus and F-actin would lead the shrink of cell bodies, condensation of cytoplasm and fragmentation of nucleus, which could be imaged by AFM to elucidate the morphological changes of apoptotic KYSE-150 cells. On the other hand, the F-actin network was thought to generate the tension inside cells and played very important roles in maintaining the biomechanics of cells, because an intact actin cytoskeleton would respond elastically to an imposed force (which was evolved into the concept of tensegrity and osmotic regulation of cells) while a fragmented cytoskeleton would lose the ability to show their native elastic behavior of cells and simply respond as an inanimate blot to be stiffer[48]. And the broken and condensation of nucleus in KYSE-150 cells induced by oridonin might also play important roles in the increased stiffness of KYSE-150 cells, because an intact nucleus of cells was also responsible for the native elasticity of cells to an applied force and the damage of nucleus thus would also induce the cells to be much stiffer. The ROS-mediated F-actin cytoskeleton structure and nucleus damage induced by oridonin therefore made KYSE-150 cells become more rigid to respond to an imposed force, making AFM force measurements an ideal method to detect the stiffness changes of KYSE-150 cells induced by oridonin. Based on these results, we demonstrated that AFM, as a powerful nanotool, could also be developed to qualitatively and quantitatively detect ROS-mediated oridonin-induced cancer cell apoptosis relying on the biophysical properties of cells.

In conclusion, the findings presented here demonstrated for the first time that AFM could be applied for the study of ROS-mediated cancer cell apoptosis based on the biophysical changes of cells. Oridonin was found to inhibit KYSE-150 cell proliferation and induce KYSE-150 cell apoptosis by inducing the over-production of intracellular ROS in a dose dependent manner, demonstrating the ROS mediated anticancer activities of oridonin in KYSE-150 cells. Based on the advantages of high resolution imaging, the morphological changes and membrane ultrastructural changes of ROS-mediated oridonin-induced KYSE-150 cell apoptosis could be determined by AFM. And by using force measurement, the biomechanical changes in ROS-mediated oridonin-induced KYSE-150 cell apoptosis could also be investigated by AFM stiffness investigation. Our findings not only provide new insights into the anticancer effects of oridonin against oesophageal cancer cells, but also highlighted the use of AFM as a powerful nanotool for qualitative and quantitative analysis of ROS-mediated cancer cell apoptosis, which could extend our understanding of the roles of ROS in cancer cell apoptosis into nanoscale.

## Supporting Information

S1 File Qualitative and quantitative analysis of ROS–mediated oridonin-induced oesophageal cancer KYSE-150 cell apoptosis by atomic force microscopy. ROS scavenger-NAC reversed oridonin induced cell cycle arrest in oesophageal cancer KYSE-150 cells (Fig A). Effects of oridonin on the adhesion force of KYSE-150 cells (Fig B). Typical force curve obtained on living KYSE-150 cells (Fig C). ROS scavenger-NAC reversed oridonin induced changes of Young’s modulus in fixed oesophageal cancer KYSE-150 cells (Fig D). ROS scavenger-NAC reversed oridonin induced cytoskeleton F-actin disruption in oesophageal cancer KYSE-150 cells (Fig E).
(DOC)

## References

[1] Gonzalez C, Sanz-Alfayate G, Agapito MT, Gomez-Nino A, Rocher A, Obeso A. Significance of ROS in oxygen sensing in cell systems with sensitivity to physiological hypoxia. Respir Physiol Neurobiol. 2002;132(1):17–41. Epub 2002/07/20. doi: S1569904802000472 [pii]. . 12126693 10.1016/s1569-9048(02)00047-2

[2] Costa A, Scholer-Dahirel A, Mechta-Grigoriou F. The role of reactive oxygen species and metabolism on cancer cells and their microenvironment. Semin Cancer Biol. 2014;25:23–32. 10.1016/j.semcancer.2013.12.007 . 24406211

[3] Ivanova D, Bakalova R, Lazarova D, Gadjeva V, Zhelev Z. The impact of reactive oxygen species on anticancer therapeutic strategies. Adv Clin Exp Med. 2013;22(6):899–908. Epub 2014/01/17. . 24431321

[4] Alexandre J, Hu YM, Lu WQ, Pelicano H, Huang P. Novel action of paclitaxel against cancer cells: Bystander effect mediated by reactive oxygen species. Cancer Research. 2007;67(8):3512–7. 10.1158/0008-5472.Can-06-3914 . 17440056

[5] Aresvik DM, Pettersen RD, Abrahamsen TG, Wright MS. 5-Fluorouracil-induced Death of Jurkat T-Cells—A Role for Caspases and MCL-1. Anticancer Research. 2010;30(10):3879–87. . 21036698

[6] Tsang WP, Chau SP, Kong SK, Fung KP, Kwok TT. Reactive oxygen species mediate doxorubicin induced p53-independent apoptosis. Life Sci. 2003;73(16):2047–58. Epub 2003/08/06. doi: S0024320503005666 [pii]. . 12899928 10.1016/s0024-3205(03)00566-6

[7] Ikezoe T, Chen SS, Tong XJ, Heber D, Taguchi H, Koeffler HP. Oridonin induces growth inhibition and apoptosis of a variety of human cancer cells. Int J Oncol. 2003;23(4):1187–93. Epub 2003/09/10. . 12964003

[8] Li CY, Wang EQ, Cheng Y, Bao JK. Oridonin: An active diterpenoid targeting cell cycle arrest, apoptotic and autophagic pathways for cancer therapeutics. Int J Biochem Cell Biol. 2011;43(5):701–4. Epub 2011/02/08. 10.1016/j.biocel.2011.01.020 S1357-2725(11)00037-9 [pii]. . 21295154

[9] Zeng R, Chen Y, Zhao S, Cui GH. Autophagy counteracts apoptosis in human multiple myeloma cells exposed to oridonin in vitro via regulating intracellular ROS and SIRT1. Acta Pharmacol Sin. 2012;33(1):91–100. Epub 2011/12/14. 10.1038/aps.2011.143 aps2011143 [pii]. 22158107 PMC4010261

[10] Gao FH, Liu F, Wei W, Liu LB, Xu MH, Guo ZY, et al Oridonin induces apoptosis and senescence by increasing hydrogen peroxide and glutathione depletion in colorectal cancer cells. Int J Mol Med. 2012;29(4):649–55. Epub 2012/02/02. 10.3892/ijmm.2012.895 22294162 PMC3577350

[11] Huang J, Wu LJ, Tashiro S, Onodera S, Ikejima T. Reactive oxygen species mediate oridonin-induced HepG2 apoptosis through p53, MAPK, and mitochondrial signaling pathways. J Pharmacol Sci. 2008;107(4):370–9. 10.1254/Jphs.08044fp . 18719315

[12] Ye LH, Li WJ, Jiang XQ, Chen YL, Tao SX, Qian WL, et al Study on the autophagy of prostate cancer PC-3 cells induced by oridonin. Anat Rec (Hoboken). 2012;295(3):417–22. Epub 2011/12/23. 10.1002/ar.21528 . 22190546

[13] Zhang YH, Wu YL, Tashiro S, Onodera S, Ikejima T. Reactive oxygen species contribute to oridonin-induced apoptosis and autophagy in human cervical carcinoma HeLa cells. Acta Pharmacologica Sinica. 2011;32(10):1266–75. 10.1038/Aps.2011.92 . 21892202 PMC4010075

[14] Chen J, Wang S, Chen D, Chang G, Xin Q, Yuan S, et al The inhibitory effect of oridonin on the growth of fifteen human cancer cell lines. Chin J Clin Oncol. 2007;4(1):16–20. 10.1007/s11805-007-0016-9

[15] Pi J, Jin H, Yang F, Chen ZW, Cai JY. In situ single molecule imaging of cell membranes: linking basic nanotechniques to cell biology, immunology and medicine. Nanoscale. 2014;6(21):12229–49. 10.1039/C4nr04195j . 25227707

[16] Jin H, Zhong X, Wang Z, Huang X, Ye H, Ma S, et al Sonodynamic effects of hematoporphyrin monomethyl ether on CNE-2 cells detected by atomic force microscopy. J Cell Biochem. 2011;112(1):169–78. Epub 2010/11/06. 10.1002/jcb.22912 . 21053362

[17] Jin H, Liang Q, Chen T, Wang X. Resveratrol protects chondrocytes from apoptosis via altering the ultrastructural and biomechanical properties: an AFM study. PLoS One. 2014;9(3):e91611 Epub 2014/03/19. 10.1371/journal.pone.0091611 PONE-D-13-46552 [pii]. 24632762 PMC3954736

[18] Kim KS, Cho CH, Park EK, Jung MH, Yoon KS, Park HK. AFM-detected apoptotic changes in morphology and biophysical property caused by paclitaxel in Ishikawa and HeLa cells. PLoS One. 2012;7(1):e30066 Epub 2012/01/25. 10.1371/journal.pone.0030066 PONE-D-11-14245 [pii]. 22272274 PMC3260205

[19] Bai HH, Jin H, Yang F, Zhu HY, Cai JY. Apigenin Induced MCF-7 Cell Apoptosis-Associated Reactive Oxygen Species. Scanning. 2014;36(6):622–31. 10.1002/Sca.21170 . 25327419

[20] Yang F, Jin H, Pi J, Jiang JH, Liu L, Bai HH, et al Anti-tumor activity evaluation of novel chrysin-organogermanium(IV) complex in MCF-7 cells. Bioorg Med Chem Lett. 2013;23(20):5544–51. Epub 2013/09/07. 10.1016/j.bmcl.2013.08.055 S0960-894X(13)00995-5 [pii]. . 24007917

[21] Pi J, Jin H, Liu R, Song B, Wu Q, Liu L, et al Pathway of cytotoxicity induced by folic acid modified selenium nanoparticles in MCF-7 cells. Appl Microbiol Biotechnol. 2013;97(3):1051–62. Epub 2012/09/05. 10.1007/s00253-012-4359-7 . 22945264

[22] Sneddon IN. The relation between load and penetration in the axisymmetric boussinesq problem for a punch of arbitrary profile. International Journal of Engineering Science. 1965;3(1):47–57. doi: 10.1016/0020-7225(65)90019-4.

[23] Radmacher M. Measuring the elastic properties of living cells by the atomic force microscope. Methods Cell Biol. 2002;68:67–90. Epub 2002/06/11. . 12053741 10.1016/s0091-679x(02)68005-7

[24] Szarama KB, Gavara N, Petralia RS, Kelley MW, Chadwick RS. Cytoskeletal changes in actin and microtubules underlie the developing surface mechanical properties of sensory and supporting cells in the mouse cochlea. Development. 2012;139(12):2187–97. Epub 2012/05/11. 10.1242/dev.073734 dev.073734 [pii]. 22573615 PMC3357912

[25] Liu JB, Yue JY. Preliminary study on the mechanism of oridonin-induced apoptosis in human squamous cell oesophageal carcinoma cell line EC9706. J Int Med Res. 2014;42(4):984–92. Epub 2014/05/31. doi: 0300060513507389 [pii] 10.1177/0300060513507389 . 24874012

[26] Pi J, Li T, Liu JX, Su XH, Wang R, Yang F, et al Detection of lipopolysaccharide induced inflammatory responses in RAW264.7 macrophages using atomic force microscope. Micron. 2014;65:1–9. 10.1016/j.micron.2014.03.012 . 25041825

[27] Laurent VM, Kasas S, Yersin A, Schaffer TE, Catsicas S, Dietler G, et al Gradient of rigidity in the lamellipodia of migrating cells revealed by atomic force microscopy. Biophys J. 2005;89(1):667–75. Epub 2005/04/26. doi: S0006-3495(05)72712-0 [pii] 10.1529/biophysj.104.052316 15849253 PMC1366565

[28] Zhang L, Yang F, Cai JY, Yang PH, Liang ZH. In-situ detection of resveratrol inhibition effect on epidermal growth factor receptor of living MCF-7 cells by Atomic Force Microscopy. Biosens Bioelectron. 2014;56:271–7. Epub 2014/02/12. 10.1016/j.bios.2014.01.024 S0956-5663(14)00027-X [pii]. . 24514079

[29] Fang YQ, Iu CYY, Lui CNP, Zou YK, Fung CKM, Li HW, et al Investigating dynamic structural and mechanical changes of neuroblastoma cells associated with glutamate-mediated neurodegeneration. Sci Rep-Uk. 2014;4. doi: Artn 7074 10.1038/Srep07074 . PMC4233341 25399549

[30] Xu L, Qu XJ, Hu XJ, Zhu ZT, Li C, Li EZ, et al Lipid raft-regulated IGF-1R activation antagonizes TRAIL-induced apoptosis in gastric cancer cells. Febs Letters. 2013;587(23):3815–23. 10.1016/j.febslet.2013.10.007 . 24161672

[31] Chaurio RA, Janko C, Munoz LE, Frey B, Herrmann M, Gaipl US. Phospholipids: key players in apoptosis and immune regulation. Molecules. 2009;14(12):4892–914. Epub 2009/12/25. 10.3390/molecules14124892 14124892 [pii]. . 20032867 PMC6255253

[32] Chapman EH, Kurec AS, Davey FR. Cell volumes of normal and malignant mononuclear cells. J Clin Pathol. 1981;34(10):1083–90. Epub 1981/10/01. 6975780 10.1136/jcp.34.10.1083 PMC494369

[33] Girasole M, Pompeo G, Cricenti A, Congiu-Castellano A, Andreola F, Serafino A, et al Roughness of the plasma membrane as an independent morphological parameter to study RBCs: A quantitative atomic force microscopy investigation. Bba-Biomembranes. 2007;1768(5):1268–76. 10.1016/j.bbamem.2007.01.014 . 17320813

[34] Demchenko AP. The change of cellular membranes on apoptosis: fluorescence detection. Exp Oncol. 2012;34(3):263–8. Epub 2012/10/17. doi: 3559 [pii]. . 23070011

[35] Song MY, Makino A, Yuan JX. Role of reactive oxygen species and redox in regulating the function of transient receptor potential channels. Antioxid Redox Signal. 2011;15(6):1549–65. Epub 2010/12/04. 10.1089/ars.2010.3648 21126186 PMC3151422

[36] Lim SC, Duong HQ, Choi JE, Lee TB, Kang JH, Oh SH, et al Lipid raft-dependent death receptor 5 (DR5) expression and activation are critical for ursodeoxycholic acid-induced apoptosis in gastric cancer cells. Carcinogenesis. 2011;32(5):723–31. Epub 2011/03/03. doi: 10.1093/carcin/bgr038 bgr038 [pii]. . 21362627 10.1093/carcin/bgr038

[37] Hossain K, Akhand AA, Kawamoto Y, Du J, Takeda K, Wu JH, et al Caspase activation is accelerated by the inhibition of arsenite-induced, membrane rafts-dependent Akt activation. Free Radical Biology and Medicine. 2003;34(5):598–606. 10.1016/S0891-5849(02)01359-X . 12614848

[38] Fang Y, Iu CY, Lui CN, Zou Y, Fung CK, Li HW, et al Investigating dynamic structural and mechanical changes of neuroblastoma cells associated with glutamate-mediated neurodegeneration. Sci Rep. 2014;4:7074 Epub 2014/11/18. 10.1038/srep07074 srep07074 [pii]. 25399549 PMC4233341

[39] Costa L, Rodrigues MS, Benseny-Cases N, Mayeux V, Chevrier J, Comin F. Spectroscopic investigation of local mechanical impedance of living cells. PLoS One. 2014;9(7):e101687 Epub 2014/07/08. 10.1371/journal.pone.0101687 PONE-D-13-43264 [pii]. 24999625 PMC4084948

[40] Radotic K, Roduit C, Simonovic J, Hornitschek P, Fankhauser C, Mutavdzic D, et al Atomic force microscopy stiffness tomography on living Arabidopsis thaliana cells reveals the mechanical properties of surface and deep cell-wall layers during growth. Biophys J. 2012;103(3):386–94. Epub 2012/09/06. 10.1016/j.bpj.2012.06.046 S0006-3495(12)00735-7 [pii]. 22947854 PMC3414883

[41] Jin H, Ma S, Song B, Ma L, Pi J, Chen X, et al Liposome impaired the adhesion and spreading of HEK293 cells: an AFM study. Scanning. 2011;33(6):413–8. Epub 2011/07/21. 10.1002/sca.20265 . 21773977

[42] Cross SE, Jin YS, Rao J, Gimzewski JK. Nanomechanical analysis of cells from cancer patients. Nat Nanotechnol. 2007;2(12):780–3. Epub 2008/07/26. 10.1038/nnano.2007.388 nnano.2007.388 [pii]. . 18654431

[43] Lekka M, Laidler P. Applicability of AFM in cancer detection. Nat Nanotechnol. 2009;4(2):72; author reply -3. Epub 2009/02/07. 10.1038/nnano.2009.004 nnano.2009.004 [pii]. . 19197298

[44] Leporatti S, Gerth A, Kohler G, Kohlstrunk B, Hauschildt S, Donath E. Elasticity and adhesion of resting and lipopolysaccharide-stimulated macrophages. FEBS Lett. 2006;580(2):450–4. Epub 2005/12/27. doi: S0014-5793(05)01506-1 [pii] 10.1016/j.febslet.2005.12.037 . 16376879

[45] Gourlay CW, Ayscough KR. A role for actin in aging and apoptosis. Biochem Soc Trans. 2005;33(Pt 6):1260–4. Epub 2005/10/26. doi: BST20051260 [pii] 10.1042/BST20051260 . 16246093

[46] Gourlay CW, Ayscough KR. The actin cytoskeleton: a key regulator of apoptosis and ageing? Nat Rev Mol Cell Biol. 2005;6(7):583–9. Epub 2005/08/03. doi: nrm1682 [pii] 10.1038/nrm1682 . 16072039

[47] Radmacher M. Measuring the elastic properties of living cells by the atomic force microscope. Atomic Force Microscopy in Cell Biology. 2002;68:67–90. . 10.1016/s0091-679x(02)68005-7 12053741

[48] Guck J, Schinkinger S, Lincoln B, Wottawah F, Ebert S, Romeyke M, et al Optical deformability as an inherent cell marker for testing malignant transformation and metastatic competence. Biophysical Journal. 2005;88(5):3689–98. 10.1529/biophysj.104.045476 . 15722433 PMC1305515

