# Supplementary material for: Qualitative and Quantitative Analysis of ROS-Mediated Oridonin-Induced Oesophageal Cancer KYSE-150 Cell Apoptosis by Atomic Force Microscopy
Source: PLoS One. 2015 Oct 23;10(10):e0140935. doi: 10.1371/journal.pone.0140935 (PMC4619704; doi:10.1371/journal.pone.0140935)
Supplement: S1 File — ROS scavenger-NAC reversed oridonin induced cell cycle arrest in oesophageal cancer KYSE-150 cells (Fig A). Effects of oridonin on the adhesion force of KYSE-150 cells (Fig B). Typical force curve obtained on living KYSE-150 cells (Fig C). ROS scavenger-NAC reversed oridonin induced changes of Young’s modulus in fixed oesophageal cancer KYSE-150 cells (Fig D). ROS scavenger-NAC reversed oridonin induced cytoskeleton F-actin disruption in oesophageal cancer KYSE-150 cells (Fig E). (DOC) [file pone.0140935.s001.doc]

**Supporting Information**


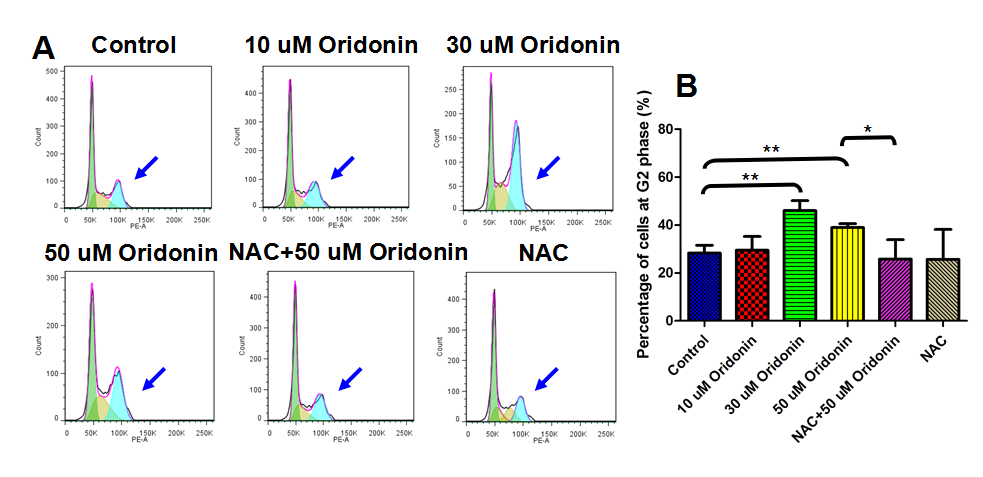


Fig. A. ROS scavenger-NAC reversed oridonin induced cell cycle arrest in oesophageal cancer KYSE-150 cells. (A) PI assay of the effects of NAC on oridonin induced cell cycle arrest in KYSE-150 cells. (B) Statistical analysis of the effects of NAC on oridonin induced cell cycle arrest in KYSE-150 cells, *p<0.05, **p<0.01. Cell cycle detection kit was used to detect the cell cycle distribution of oridonin treated KYSE-150 cells according to the manufacturer’s instructions. The cells were seeded into 6 well plates with a density of 1×10^5^ cells/well for 24 h and incubated with different concentration of oridonin for 24 h. To scavenge the ROS produced by oridonin, cells were pretreated with 2.5 mM NAC for 1 h and then treated with oridonin for 24 h. After treatment with oridonin, cells were harvested, washed with PBS, and fixed with 70 % ethanol overnight at 4 °C. The fixed cells were washed three times with PBS, treated with RNase A, stained with PI (50 μ g/mL) for 30 min at 37°C, and analyzed by a flow cytometry (BD, USA).


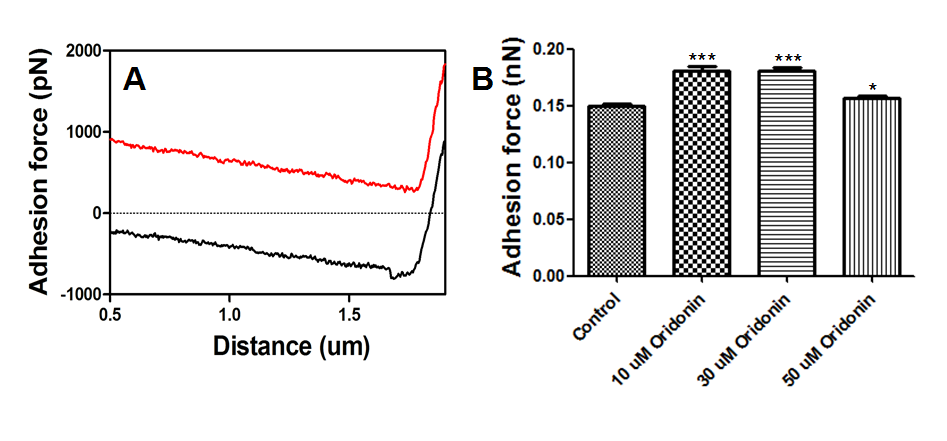


Fig.B. Effects of oridonin on the adhesion force of KYSE-150 cells. (A) Typical adhesion force curves show no adhesion events (Red one) and strong adhesion events (Black one). (B) Statistical analysis of the effects of oridonin on the adhesion force between AFM tip and KYSE-150 cells, n>3500, *p<0.05, ***p<0.001. The adhesion force curves between AFM tips and KYSE-150 cells were also determined by Force Volume of AFM similar with the procedures for AFM nanoindentation and stiffness analysis. The adhesion force in each adhesion force curve was analyzed by the instrument equipped Nanoscope analysis software.


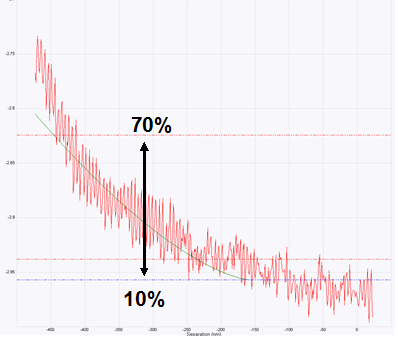


Fig.C. Typical force curve obtained on living KYSE-150 cells: experimental data (red line) and fitted data generated by Sneddon model (green line), the fitting processes of force curves were directly provided by the Nanoscope Analysis Software. For stiffness calculation, the min and max force fit boundary were set as 10% and 70% of the indentation curves, which meant that the fitted curves (green line) between the blue line and the upper red line were used for stiffness calculation.


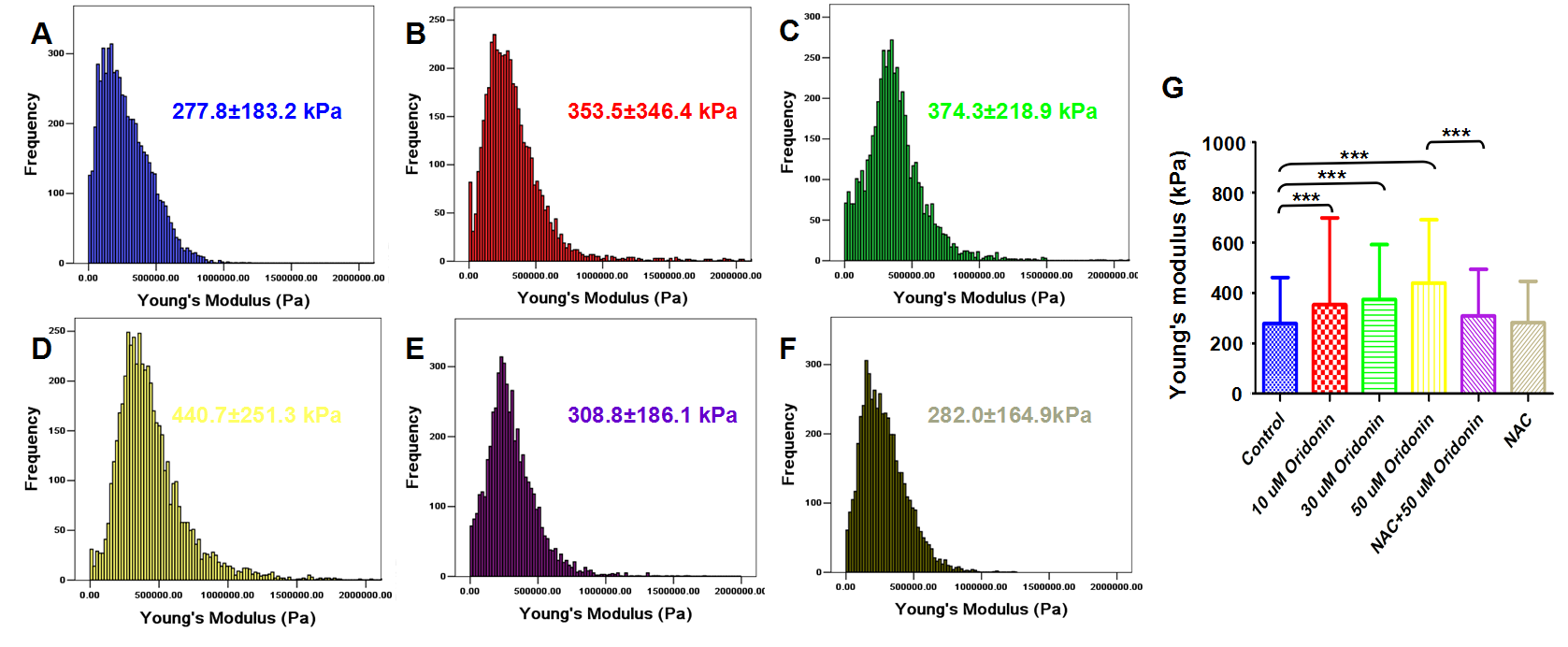


Fig.D. ROS scavenger-NAC reversed oridonin induced changes of Young’s modulus in fixed oesophageal cancer KYSE-150 cells. (A) Histogram distribution of Young’s modulus obtained from KYSE-150 cell. (B) Statistical analysis of the effects of NAC on oridonin induced KYSE-150 cell Young’s modulus changes, results were obtained on 20 different cells, n>5000, ***p<0.001. Biomechanical properties of fixed KYSE-150 cells were measured in PBS solution by bare tips in Force Volume mode through AFM (Bruker, German). The spring constant of silicon nitride probes used for fixed cell measurements (BudgetSensors, Bulgaria) was calibrated by the thermal-noise method in a clean culture dish containing PBS, which was 1.08±0.08 N/m with a deflection sensitivity of 13.79±0.56 nm/V. In the Force Volume mode, the tip was alternately approached to cell surface and then retracted from 16×16 points over 1×1 um^2^ area on sample surface while force curves were synchronously recorded. More than 20 different locations on 20 different cells at the central area of cells (The highest area of cells) were recorded in each group. The Young’s modulus was calculated from the force curves by basic Sneddon model, which described the behavior of a known geometry indenter in contact with an elastic half-space much less rigid than the punch as shown in equation (1):

$F=\frac{2}{\pi}\text{t}an(\alpha)\frac{E}{(1-\nu^{2})}\delta^{2}$ (1)

Where υ, F, δ, E, and α are Poisson ratio, loading force, indentation, Young’s modulus, and the half-opening angle of a conical tip, respectively. In Sneddon model, the load exerted by the punch is linked to the caused indentation depth, which shows the relationship between the applied force F and the indentation δ in the case of conical indenter. A Poisson ratio of 0.5 is appropriate for cells and thus used in the following analysis.


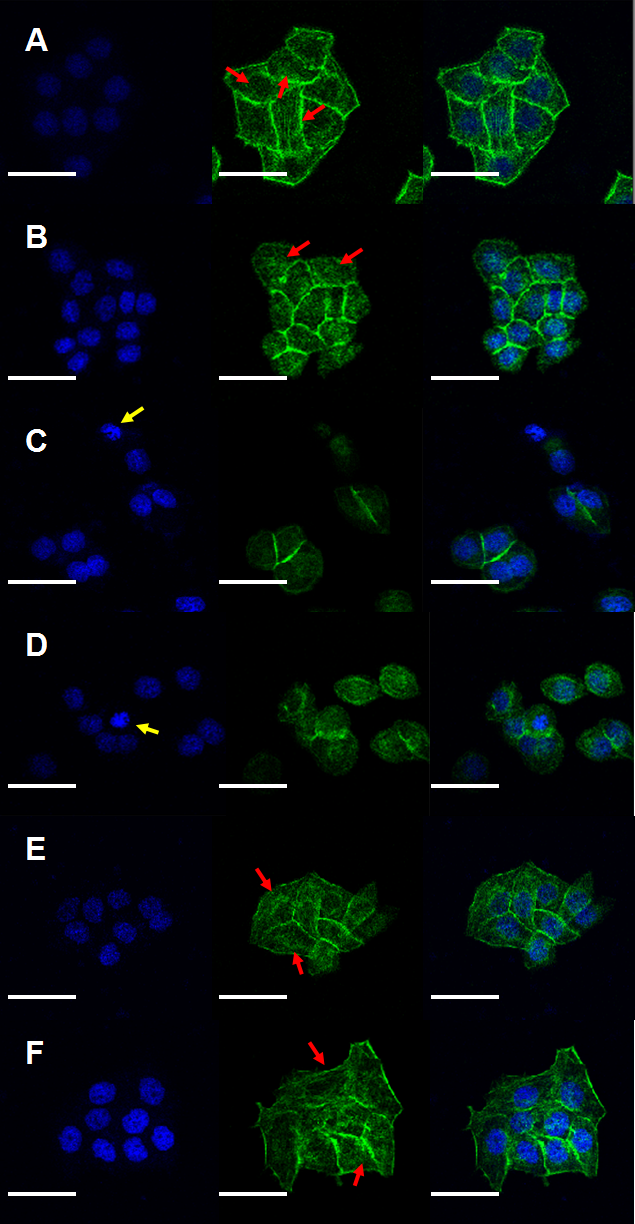


Fig.E. ROS scavenger-NAC reversed oridonin induced cytoskeleton F-actin disruption in oesophageal cancer KYSE-150 cells. Confocal microscopy imaging of F-actin (Left panel), nucleus (Middle panel) and merged (Right panel) in (A) control, (B) 10 μM oridonin treated, (C) 30 μM oridonin treated, (D) 50 μM oridonin treated, (E) 2.5 mM NAC+50 μM oridonin treated and (F) 2.5mM NAC treated KYSE-150 cells, scale bar: 50 μm. Red arrows indicate the thread like F-actin fibres and yellow arrows indicated the broken or condensation of nucleus. The structure of F-actin and nucleus in KYSE-150 cells was investigated by confocal microscopy imaging with special staining of F-actin with actin-tracker green and nucleus with DAPI. After treated with oridonin for 24 h, KYSE-150 cells were fixed by 4 % paraformaldehyde for 10 min, washed with PBS (1% TritonX 100) and then incubated with 200 μL actin-tracker green for 60 min in dark at room temperature. After washed triple with PBS, cells were then incubated with 50 μM DAPI for 4 min and washed triple with PBS. Confocal microscopy (Leica, German) was used to image the nuclear morphology and the organization of F-actin cytoskeleton structure.
